# Quality analysis of YouTube videos on vulvodynia

**DOI:** 10.1093/sexmed/qfac013

**Published:** 2023-01-12

**Authors:** Savas Ozgur Aglamis, Samet Senel, Antonios Koudonas

**Affiliations:** Obstetrics and Gynecology Clinic, Istanbul 34371, Turkey; Urology Clinic, Ankara City Hospital, Ankara 06800, Turkey; First Department of Urology, Faculty of Medicine, Aristotle University of Thessaloniki, Thessaloniki 54124, Greece

**Keywords:** DISCERN, GQS, quality, vaginismus, YouTube

## Abstract

**Background:**

Social media, with its low cost and easy accessibility, represents an ideal medium for sharing medical interests, but the quality of its content is questionable.

**Aim:**

The primary aim of this study was to evaluate the quality of video content related to vaginismus on YouTube as a source of information by using scores of established classification systems. The secondary aim was to examine the relationship between objective and subjective measures of their quality.

**Methods:**

The term *vaginismus* was typed into the YouTube search bar (http://www.youtube.com). The first 50 videos with the highest number of views were included in the study. All videos were assessed on August 18, 2022, by a gynecologist and/or a urologist experienced in vulvodynia. Data of all the videos were recorded, such as source, content, duration, day since upload date, number of views, number of likes, number of comments, and views per day. The Global Quality Scale (GQS) and modified DISCERN score were employed to assess the quality of the videos.

**Outcomes:**

The primary outcomes of this study were the scores of established classification systems and the measures relating to the preference and evaluation of viewers of YouTube videos on vulvodynia.

**Results:**

A total of 50 videos were evaluated. The sources of 32 (64%) of these videos were “universities/professional organizations/nonprofit physicians/physicians” and “stand-alone health information websites.” The GQS and modified DISCERN scores of the videos whose source was “universities/professional organizations/nonprofit physicians/physicians” were higher than those whose source was “talk show programs/television programs” (*P* = .014 for GQS score, *P* = .046 for modified DISCERN score). When classified according to GQS score, the quality level of 58% of the videos was low. Of the videos whose source was “universities/professional organizations/nonprofit physicians/physicians,” 56.3% were of good quality.

**Clinical Implications:**

The quality of information was very low and suggested that health care professionals should take on a more active role in configuring the qualitative characteristics of the material available online about the given health issues.

**Strengths and Limitations:**

To the best of our knowledge, this is the first study to investigate the quality of YouTube videos on vaginismus (vulvodynia). However, the limitation of this study is the relatively subjective evaluation of videos, including the risk of observer bias, although we attempted to overcome this problem with the inclusion of 2 independent reviewers and the use of validated tools.

**Conclusion:**

YouTube videos may offer a huge amount of information about this condition, but the quality of the available sources is heterogeneous.

## Introduction

Besides the detrimental effect of vulvodynia on female sexual function, several additional implications with a decisive effect on the psychosomatic well-being of the patient have been recorded: a diminished self-concept regarding the role as a woman, increased psychological disorders (anxiety, depression, and others), a tendency for selection of cesarean section, avoidance of gynecologic examination, fertility disorders, and dysfunctional relational status in the frame of a marriage or relationship.^1^ Given the definitional discrepancies and the reluctance of the patient to apply for specialized consultation, the actual prevalence rate has not been estimated yet.^2^

The range of the recorded prevalence depends on the level of estimation, varying from 0.5% to 1% in the community to 42% in specialized centers of female sexual disorders. Regarding the etiology of vulvodynia, there is a consensus on the psychophysiologic origin of the problem. Psychological factors—including unpleasant experiences, negative sexual attitudes combined with a lack of sexual education, and negative feedback in the frame of a dysfunctional relationship—represent the main etiologic factors.^3^ Regarding the organic factors, a minority of vulvodynia cases can be attributed to a number of pathologies, such as congenital abnormalities, infections, previous surgery or radiotherapy, vaginal atrophy, or dysfunctional pelvic floor musculature.^3^ Regardless of the initial etiologic factor, the onset of vulvodynia symptomatology is perpetuated through catastrophizing thinking and negative expectations, which reinforce the psychological burden on the patient, forming the “vicious cycle of vulvodynia.” Besides the stated mechanism, which constitutes the intrapersonal dimension of vulvodynia, the role of the interpersonal dimension seems to be equally important, since a dysfunctional relationship may also intensify the manifestation of vulvodynia symptoms.^4^

Due to the biopsychosocial nature of vulvodynia, priority is given to the methods of reeducating and informing female patients about sexual function and its aberration in the case of vulvodynia. Social media represents an ideal medium for sharing information based on medical interests, given its low cost and wide reach, yet it remains underused by health professionals.^5^ This emerging form of sharing health information is characterized by special patterns of effect across the population. Indeed, it seems that mostly younger adults utilize social media and are in the position to benefit from the presented information.^6^ The main source of mistrust seems to be the absence of validated quality indexes of the online material. A review comparing a number of studies evaluating medical YouTube videos concluded that in 2015 there was no concrete set of evaluation criteria and every included study was based on a devised scoring system.^7^ This situation creates an obstacle for analysis duplication and the establishment of a systematic process, which can play an objective regulating role in the interaction with the patient. Another review examined the publications addressing the health care information on YouTube and concluded that YouTube is an emerging medium for disseminating health information; however, it may contain misleading information or be used for the promotion of unscientific therapies. Another conclusion was that the sources related to government organizations and professional associations contain the most trustworthy and high-quality material.^8^ In the era of established assessment tools for the reliability of medical videos, a recent review examined articles on the use of YouTube for assessing medical information.^9^ The study found that the majority of the studies used assessment tools and many videos contained misleading information. Moreover, 36.3% of the videos were uploaded by trusted sources, such as official organizations and health professionals, while 63.7% were produced from sources with nonverifiable affiliations.^9^ Importantly, only 35% of the studies strongly recommended the implementation of medical videos as a reliable source of health information.

The online database of audiovisual materials on health and public information is constantly growing. The quality of this material is considered heterogeneous. For this reason, we aimed to evaluate the quality of existing YouTube videos about vulvodynia. Our study recorded objective measures of quality, such as the scores of established classification systems, and subjective measures of quality, such as those relating to the preference and evaluation of the viewers. Subsequently, we performed analyses about the association of quality with specific characteristics of the videos.

The questions that this study tries to answer are as follows: Does the information that patients learn about vulvodynia from social media reflect the truth? Is the quality level of these videos satisfactory?

## Methods

For this study, ethical approval was obtained from the local ethics committee (E2-22-2268).

Although there has been an update in nomenclature in regard to vulvodynia as of 2015, the term *vaginismus* was typed into the YouTube search bar (http://www.youtube.com) because the target population of the present study is patients, who are not health professionals and who prefer using that term. As a result of the unfiltered search, the videos that had no sound, were not in English, and were repetitive were excluded from the study. The same videos uploaded from the same source at different times were considered “repetitive videos,” and the old ones were included in the study. In total, 77 videos were viewed. The first 50 videos with the highest number of views were included in the study.

All videos were reviewed and assessed on August 18, 2022, by a gynecologist and/or a urologist who was experienced in vulvodynia. Two physicians separately reviewed and scored all videos according to the Global Quality Scale (GQS) and modified DISCERN score. For each video, if the score determined by both physicians was the same, the video was scored as such. If the physicians set a different score for the same video, they rewatched it and determined a common score. Data from all the videos were recorded:

*Source:* universities/professional organizations/nonprofit physicians/physicians, stand-alone health information websites, medical advertisements/for-profit organizations, talk show programs/television programs

*Content:* general information, symptoms and diagnosis methods, treatments, side effects, lifestyle/diet, other

*Time:* Duration in seconds and day since upload date

*Frequency:* number of views, likes, comments, and views per day since upload date

The GQS and modified DISCERN score were employed to assess the quality of the videos.^10^^,^^11^

The GQS was developed by Bernard et al in 2007 to assess video quality. For this purpose, the flow, quality, and usefulness properties of videos are evaluated in this scoring system, and videos are scored between 1 and 5 points:

*Score 1:* Poor quality, poor flow of the site, most information missing, not at all useful for patients.

*Score 2:* Generally poor quality and poor flow, some information listed but many important topics missing, of very limited use to patients.

*Score 3:* Moderate quality, suboptimal flow, some important information adequately discussed but others poorly discussed, somewhat useful for patients.

*Score 4:* Good quality and generally good flow, most of the relevant information listed but some topics not covered, useful for patients.

*Score 5:* Excellent quality and excellent flow, very useful for patients.

According to this system, videos with a score of 1 and 2 points are classified as low quality, those with 3 points as intermediate, and those with 4 and 5 points as good.^10^

The DISCERN scoring system was developed by Charnock et al in 1999. It consists of 15 questions and assesses the quality of the information.^11^ In 2012, Singh et al used its modified form with 5 questions:

- Are the aims clear and achieved?
- Are reliable sources of information used (ie, publication cited)?
- Is the information presented balanced and unbiased?
- Are additional sources of information listed for patient reference?
- Are areas of uncertainty mentioned?

According to this scoring system, each question is scored as 1 point (if the answer ise `yes') or 0 point (if the answer ise `no'). So, each video is scored between 0–5 points in total.

### Statistical analysis

Data coding and statistical analyses were performed through SPSS version 22 (IBM). The Shapiro-Wilk test was used to examine whether the variables were normally distributed. The Kruskal-Wallis test was employed to evaluate the difference among medians. In cases where >2 variables were compared, Bonferroni correction was used to determine which variable caused the difference. Correlation between variables was analyzed with the Spearman test. A *P* value <.05 was considered statistically significant.

## Results

A total of 50 videos were evaluated. Sources of 32 (64%) of these videos were “universities/professional organizations/nonprofit physicians/physicians” (n = 16) and “stand-alone health information websites” (n = 16). The content of 20 (40%) videos was about “general information.” The median number of views of the videos was 10 748, and the median duration of view was 343 seconds. The median GQS score was 2 and the modified DISCERN score was 3. Table 1 shows the content, features, and quality scores of the videos.

**Table 1:** Features and quality scores of the videos.

	No. (%) or median (range)
Sources of the videos	
Universities/professional organizations/nonprofit physicians/physicians	16 (32)
Stand-alone health information websites	16 (32)
Medical advertisements/for-profit organizations	7 (14)
Talk show programs/television programs	11 (22)
Content of the videos	
General information	20 (40)
Symptoms and diagnosis methods	3 (6)
Treatments	15 (30)
Side effects	1 (2)
Lifestyle/diet	4 (8)
Others	7 (14)
Features of the videos	
Duration, s	343 (32-5576)
Days since upload date, d	907 (118-3133)
No. of views	10 728 (287-391 414)
No. of likes	95 (0-6712)
No. of comments	3 (0-425)
View per day	12.1 (0.1-1104.6)
Quality scores of the videos	
Global Quality Scale	2 (1-5)
Modified DISCERN	3 (1-5)

The quality scores of the videos, divided according to their sources, were significantly different from one another (*P* < .05). According to the result of the Bonferroni correction, the GQS and modified DISCERN scores of the videos whose source was “universities/professional organizations/nonprofit physicians/physicians” were higher than those whose source was “talk show programs/television programs” (4 vs 1 for GQS score, *P* = .014; 4 vs 2 for modified DISCERN score, *P* = .046; Table 2).

**Table 2:** Quality assessment of the videos according to the source of videos.

	Quality scores of the videos, median (range)	
Sources of the videos	Global Quality Scale	Modified DISCERN
Universities/professional organizations/nonprofit physicians/physicians	4 (1-5)	4 (1-5)
Stand-alone health information websites	2 (1-4)	2.5 (1-4)
Medical advertisements/for-profit organizations	2 (1-5)	2 (2-5)
Talk show programs/television programs	1 (1-5)	2 (1-5)
*P* value^a^	**.012**	**.045**

^a^Bold indicates *P* < .05.

When classified according to the GQS score, the quality level was low for 58% of the videos. However, 56.3% of the videos were of good quality when their source was “universities/professional organizations/nonprofit physicians/physicians.” The median (range) number of views was 7016.5 (287-114 695) for low-quality videos, 53 401 (4507-182 926) for intermediate quality, and 72 852 (1110-391 414) for high quality. The median (range) duration of videos was 281 seconds (32-4229) for low-quality videos, 377 (202-5576) for intermediate quality, and 299 (116-1826) for high quality. According to this classification, the median numbers of views, likes, comments, and views per days were higher in the videos with good and intermediate quality than those with low quality (*P* < .05). There was no statistical difference between good- and intermediate-quality videos in terms of these parameters (*P* > .05). In addition, no statistically significant difference was found among the videos by quality groups in terms of duration and days since upload date (*P* > .05; Table 3).

**Table 3:** Sources and features of the videos classified according to GQS score.

	Classification of GQS score			
Videos	Low	Intermediate	Good	*P* value^a^
**Source, No. (%)**				
Universities/professional organizations/nonprofit physicians/physicians	4 (25)	3 (18.7)	9 (56.3)	
Stand-alone health information websites	11 (68.7)	4 (25)	1 (6.3)	
Medical advertisements/for-profit organizations	5 (71.4)	1 (14.3)	1 (14.3)	
Talk show programs/television programs	9 (81.8)	1 (9.1)	1 (9.1)	
**Feature, median (range)**				
Duration, s	281 (32-4229)	377 (202-5576)	299 (116-1826)	.367
Days since upload date	937 (118-3133)	694 (281-1957)	870 (393-2003)	.347
No. of views	7016.5 (287-114 695)	53 401 (4507-182 926)	72 852 (1110-391 414)	**.006**
No. of likes	21.5 (0-213)	730 (151-6712)	2165 (782-3200)	**<.001**
No. of comments	1 (0-45)	25 (1-147)	86 (0-425)	**<.001**
Views per day	8.4 (0.1-1104.6)	60.7 (3.7-190)	72.7 (0.6-996)	**.003**

Abbreviation: GQS, Global Quality Scale.

^a^Bold indicates *P* < .05.

According to the correlation analysis, the GQS and modified DISCERN scores were correlated (*P* < .001). Again, a positive correlation was found between both scoring systems and numbers of views, likes, comments, and views per day (*P* < .05). No correlation was found between duration and days since upload date and the scoring systems (*P* > .05; Table 4).

**Table 4:** Correlation analyses for GQS and modified DISCERN scores of the videos.

	GQS score		Modified DISCERN score	
	*r*	*P* value^a^	*r*	*P* value^a^
GQS score			0.831	**<.001**
Modified DISCERN score	0.831	**<.001**		
Duration, s	0.037	.801	0.054	.71
Days since upload date	−0.143	.323	−0.251	.079
No. of views	0.43	**.002**	0.342	**.015**
No.of likes	0.685	**<.001**	0.652	**<.001**
No.of comments	0.517	**<.001**	0.462	**.001**
Views per day	0.4	**.004**	0.372	**.008**

Abbreviation: GQS, Global Quality Scale.

^a^Bold indicates *P* < .05.

## Discussion

Vulvodynia represents an underresearched sexual disorder with universal prevalence whose ideal treatment remains to be documented on objective data and defined. Vulvodynia is a disorder with a psychosocial nature; as such, the initiatives for informing the female population about the etiology and the therapeutic perspectives are expected to have an important role in the improvement of therapeutic results. A major part of this public policy is represented by the informative material available online. In this study, we aimed to evaluate the quality of the most viewed videos on vulvodynia. Our results showed that the majority of videos were produced by official health-related organizations and health care professionals and contained specific information about vulvodynia. The quality classification of the majority of videos was low, which was not representative of the videos with official organization origin. The quality scores achieved from the videos produced by official organizations were significantly higher than the videos from talk shows/television programs. Videos classified as having high and intermediate quality had a significantly higher number of views, views per day, likes, and comments as compared with videos of low quality. High- and intermediate-quality videos did not differ in terms of the aforementioned parameters. Moreover, the duration was not associated with the quality classification of the videos. Regarding the correlation analysis, the GQS and modified DISCERN scores were strongly correlated, which serves as a surrogate for their validity as assessment tools. Furthermore, a strong correlation was found between standardized quality classification and the number of likes, which reflects the agreement between the classification systems used in the study and the perceived video quality from the public. A moderate correlation was found between standardized classification and the numbers of views, views per day, and comments. All stated correlations were statistically significant.

The current results accord with the available data about medical videos in general and the limited data about vulvodynia-related videos. Moreover, the videos uploaded by health professionals were more reliable and were proposed as the ideal medium for informing the patients. Another recent report evaluated websites containing information about pain-inducing sexual female disorders and used standardized questionnaires for quality classification.^12^ The study concluded that the quality of information was very low and suggested that health care professionals should take on a more active role in configuring the qualitative characteristics of the material available online about the aforementioned health issues.

In our opinion, the importance of the role of online sources in reducing the burden of suffering associated with vulvodynia and other female pain-inducing sexual disorders is already recognized. A randomized protocol is currently examining the effect of internet-based guided self-help intervention in comparison with a control group.^13^ A review of the existing mobile applications focused on sexual health found an application specialized for pain-inducing female sex disorders.^14^ The available online and mobile information sources are expected to not only contribute to a better understanding of the psychophysiological background of vulvodynia but also encourage more women to apply for specialized consultation. This need for professional treatment frequently remains unexpressed in the frame of cultural, personal, and environmental factors and ignorance about the consequences of the condition and the existing therapeutic perspectives.

In conclusion, vulvodynia represents an underresearched health issue, which in the modern era can be treated or at least modified with the assistance of internet sources. YouTube videos may offer a huge amount of information about this condition, but the quality of the available sources is heterogeneous. Health professionals should address the qualitative issues of the material available online and configure a more reliable, updated, and informative online environment to maximize its effect on reducing the burden of vulvodynia-related suffering.
